# NMR spectra of PB2 627, the RNA-binding domain in influenza A virus RNA polymerase that contains the pathogenicity factor lysine 627, and improvement of the spectra by small osmolytes

**DOI:** 10.1016/j.bbrep.2017.09.003

**Published:** 2017-09-20

**Authors:** Yusuke S. Kato, Masaru Tanokura, Takashi Kuzuhara

**Affiliations:** a Institute for Health Sciences, Tokushima Bunri University, Tokushima 770-8514, Japan; b Department of Applied Biological Chemistry, Graduate School of Agricultural and Life Sciences, University of Tokyo, Tokyo 113-8657, Japan; c Institute for Enzyme Research, Tokushima University, Tokushima 770-8503, Japan; d Faculty of Pharmaceutical Sciences, Tokushima Bunri University, Tokushima 770-8514, Japan

**Keywords:** CHAPS, 3-[(3-cholamidopropyl) dimethylammonio]-1-propanesulfonate, DTT, dithiothreitol, Irel, ratio of signal intensity to noise level, HSQC, heteronuclear single quantum coherence, NDSB, non-detergent sulfobetaine, NMR, nuclear magnetic resonance, PB2 627, C-terminal RNA-binding domain of PB2 containing lysine 627, S/N, signal-to-noise ratio, TMAO, trimethylamine N-oxide, Influenza A virus, RNA polymerase, PB2 627, NMR, additive

## Abstract

The influenza A virus, which has an RNA genome, requires RNA-dependent RNA polymerase for transcription and replication. The polymerase is comprised of the subunits PA, PB1, and PB2. The C-terminal RNA-binding domain in PB2 contains lysine 627 (PB2 627), which is associated with pathogenicity and host range. However, the structure and molecular mechanism of PB2 627 in solution remain obscure. Here, we investigated PB2 627 in solution by nuclear magnetic resonance (NMR) and detected inhomogeneity in the intensities of backbone amide proton signals due to local fluctuations in structure. To characterize the effects of chemical chaperones on spectral data and improve the data quality, we tested 20 different additives, including L-arginine L-glutamate salt, (L-arginine)_2_SO_4_, glycerol, β-octylglucoside, 3-[(3-cholamidopropyl) dimethylammonio]-1-propanesulfonate, Na_2_SO_4_, 1,5-diaminopentane, 1,4-diaminobutane, trehalose, sucrose, glycine, trimethylamine N-oxide, β-alanine, L-α-alanine, hydroxyectoine, betaine, L-proline, and non-detergent sulfobetaine 195, 201, and 256. We evaluated the quality of the resulting spectra by calculating the standard deviation and average of the ratio of signal intensities to noise level of amide peaks, as well as the ratio of the standard deviation to the average. NMR-profile analysis revealed diverse effects of additives on the dynamic properties of PB2 627. Based on such criteria, we found that small osmolytes such as glycine and L-α-alanine reduced structural fluctuations and improved the quality of spectral data, which is likely to facilitate a detailed NMR-based structural analysis. The methodology developed here may also be more generally useful for evaluating the effects of chemical chaperones on the structural integrity of proteins.

## Highlights

• The NMR spectrum of PB2 627 showed well dispersed signals.
• Its signal intensity was inhomogeneous, suggesting structural fluctuations.
• Glycine and L-α-alanine reduced structural fluctuations.
• Glycine and L-α-alanine improved the quality of spectral data of NMR.

## 1 Introduction

In 1918, a pandemic of influenza A virus resulted in ten million deaths worldwide [1]. Strategies to prevent future pandemics must therefore be formulated [2], [3]. Although inhibitors of the viral neuraminidase and M2 ion channel are widely used for treatment [4], [5], some adverse side effects have been reported, and drug resistance has emerged [6], [7]. The viral RNA-dependent RNA polymerase is a very promising alternative drug target because it is required for transcription and replication of viral genes. To initiate viral gene transcription, a 5′ RNA fragment of 10–15 nucleotides is used as a primer [8], which is recognized by the C-terminal domain in the PB2 subunit of the RNA polymerase along with promoter RNA [9]. This domain also contains the lysine 627 residue, which is associated with high pathogenicity and restricted host range. This domain is thus called PB2 627 [10].

Protein tertiary structures are a valuable resource in drug development, and crystal structures of PB2 627 have been determined [11], [12], [13]. Nevertheless, it is equally important to investigate the structure and dynamics of PB2 627 in solution in order to understand its mechanism of action, and to design novel drugs against it. In turn, such an investigation would require optimal conditions for solution nuclear magnetic resonance (NMR). In this study, we collected 2D NMR spectra from PB2 627 and found that the quality of the initial spectra is unlikely to be sufficient for higher-dimensional NMR, which is necessary to analyze structure and dynamics, but has a lower signal-to-noise ratio (S/N) than 2D NMR.

Previous studies demonstrated that diverse additives, such as salts, sugars, amino acids, methylamines, chaotropic agents, and detergents, improved NMR spectra of proteins [14], [15]. L-arginine L-glutamate salt and non-detergent sulfobetaines (NDSBs) are frequently used to this end [16], [17]. Diverse amino acids have been reported to improve protein stability [18], [19], [20]. Diamines were reported to prevent thermal aggregation and inactivation of lysozyme [21]. Amino acids, methylamines, and chaotropes are categorized as osmolytes. Osmolytes are osmotically active solutes in organisms that have evolved to resist water stress such as high or low salt concentrations, desiccation, or freezing [22]. Most osmolytes are small organic compounds. However, to our knowledge, no reports have described the comprehensive testing of numerous additives and their effects on protein NMR spectra. Thus, we experimented with 20 different compounds to improve the spectra of PB2 627, finding that osmolyte additives such as neutral amino acids and derivatives can improve the quality of spectral data. We anticipate that our results and methodologies will prove generally useful for improving protein NMR spectra and for evaluating the ability of various compounds to reduce structural fluctuations of proteins.

## 2 Materials and methods

### 2.1 Sample preparation

Construction of a plasmid for expressing His_6_-tagged PB2 627 (amino acids 535–759) was described previously [11], [12]. ^15^N-labeled PB2 627 was produced in Rosetta 2 (DE3) *E. coli* (EMD Millipore, Billerica, MA), following a protocol based on M9 media containing trace elements [23], with some modification. Briefly, cells were lysed by sonication in 20 mM sodium phosphate (pH 7.6), 250 mM NaCl, 7.5% glycerol, 20 mM imidazole pH 8.0, 1 mM dithiothreitol (DTT), protease inhibitor cocktail (Sigma-Aldrich, St. Louis, MO), and lysozyme (Seikagaku Corp., Tokyo, Japan). Lysates were clarified by centrifugation at 50,000 × *g*, and the supernatant was loaded on Ni-NTA agarose (Qiagen, Hilden, Germany) equilibrated with 20 mM sodium phosphate (pH 7.3), 250 mM NaCl, 5% glycerol, and 20 mM imidazole (pH 8.0). After extensive washing, PB2 627 was eluted with 20 mM sodium phosphate (pH 7.0), 250 mM NaCl, 1 mM DTT, 10% glycerol, and 200 mM imidazole (pH 8.0). After addition of 0.1% Tween 20 and thrombin (GE Healthcare Bio-Sciences Corp., Piscataway, NJ), the protein was dialyzed against 20 mM Tris-HCl (pH 6.5), 50 mM NaCl, 10% glycerol, and 0.5 mM DTT, and then purified by cation-exchange on HiPrep CM (GE Healthcare). Subsequently, the protein was concentrated using a Vivaspin centrifugal concentrator (Sartorius, Goettingen, Germany) and loaded on Superdex 75 16/600 (GE Healthcare) equilibrated with 20 mM sodium phosphate (pH 6.5), 150 mM NaCl, 4% glycerol, and 1 mM DTT. Finally, purified samples were concentrated and dialyzed against 20 mM sodium phosphate (pH 6.5), 7% D_2_O, 50 mM NaCl, and 1 mM DTT.

### 2.2 NMR

^1^H–^15^N heteronuclear single quantum coherence (HSQC) experiments were performed at 20 °C using an Inova 600 spectrometer equipped with a Cold Probe (Agilent Technologies, Santa Clara, CA) and an Avance-III 950 system (Bruker, Billerica, MA). The final concentration of PB2 627 was 3.0 mg/mL, although PB2 627 is stable and can be crystallized at concentrations of up to 10 mg/mL [11]. Spectra were collected with or without 0.2 M (L-arginine)_2_SO_4_, Na_2_SO_4_ or L-arginine L-glutamate salt; 4% glycerol; 25 mM β-octylglucoside; 10 mM CHAPS; 0.1 M 1,5 diaminopentane or 1,4 diaminobutane; 20% trehalose; or 0.5 M sucrose, glycine, trimethylamine N-oxide (TMAO), β-alanine, L-α-alanine, hydroxyectoine, betaine, L-proline, or NDSB 195, 201, or 256 (Supplementary Table 1). NMR tubes were siliconized prior to use to prevent protein aggregation. Data processing and analysis were performed with the NMRpipe and Sparky 3.115 programs [24], [25]. Relative intensity (I_rel_) denotes the ratio of signal intensity to noise level according to Pedrini et al. [26].

## 3 Results

### 3.1 Flash 2D NMR

PB2 627 contains a nuclear localization signal, a C-terminal RNA-binding domain, and lysine 627 that determines the pathogenicity and host range. We purified PB2 627 to homogeneity using immobilized Ni-affinity, cation-exchange, and gel-filtration chromatography (Supplementary Fig. 1).

We carried out a ^1^H–^15^N heteronuclear single quantum coherence experiment by NMR. Fourier transformation and processing were then performed to obtain a spectrum. The spectrum contained numerous sharp, well-dispersed peaks from backbone amides (Supplementary Fig. 2A), suggesting that PB2 627 is monomeric, and that the global fold is stable in aqueous solution. However, the number of peaks diminished when the spectrum was contoured at a higher threshold (Supplementary Fig. 2B), indicating that the peaks had different intensities. Weak amide signals arise from line broadening due to local conformational changes over a time scale ranging from microseconds to milliseconds. Structural polymorphisms and transient oligomerization/aggregation of proteins can also cause weak signals. In contrast, strong amide signals arise from unstructured regions. Thus, inhomogeneity in signal intensities is due to inhomogeneity in structure and structural dynamics of proteins. Indeed, the PB2 627 construct contains a C-terminal segment that was not observed in the crystal structure. This presumably unstructured region should provide strong signals at 8.0–8.5 ppm of the ^1^H axis in the HSQC spectrum. It is therefore likely that structural analysis using this sample will be challenging. In addition, we anticipate that many amides with low-intensity peaks will become undetectable in higher-dimensional NMR, which has a lower S/N than does HSQC.

### 3.2 Effects of additives

To reduce inhomogeneity in the intensity of amide peaks, we investigated 20 different additives that may homogenize the structure and dynamic properties of PB2 627 and improve the quality of NMR data. We tested the diamines 1,4-diaminobutane, and 1,5-diaminopentane; the detergents β-octylglucoside and CHAPS; the sugars and polyols glycerol, trehalose, and sucrose; the salt Na_2_SO_4_; NDSBs 195, 201, and 256; and the amino acids and amino-acid derivatives hydroxyectoine, (L-arginine)_2_SO_4_, L-arginine L-glutamate salt, TMAO, betaine, L-proline, L-α-alanine, β-alanine and glycine. Prior to investigating the effects of these additives on amide peak homogeneity, we studied different salt concentrations and pH values, which were not effective.

**Fig. 1:**
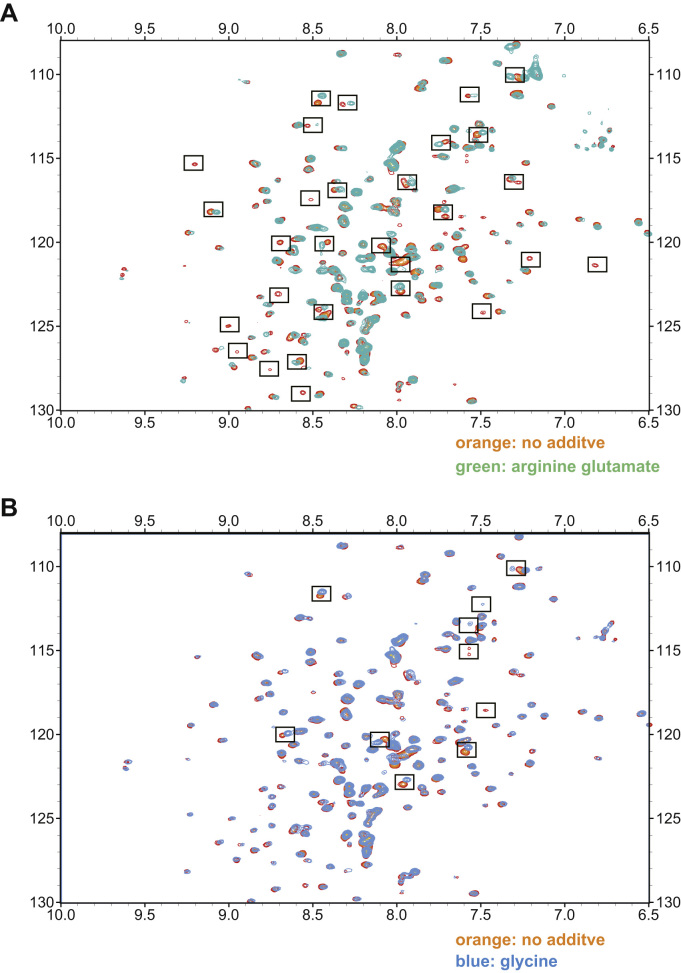
^**1**^**H-**^**15**^**N HSQC spectra with or without additives** Overlay of PB2 627 spectra without additives and (A) with L-arginine L-glutamate salt or (B) with glycine. The boxed peaks were markedly moved.

HSQC spectra of sufficient quality for further analysis were obtained in the presence of 17 different additives (Supplementary Fig. 3). Spectra collected in the presence of β-octylglucoside, sucrose, and NDSB 256 were not suitable for further analysis, presumably because PB2 627 was denatured or aggregated under these conditions. Spectra obtained in the presence of L-arginine L-glutamate salt and glycine represented examples with contrastive spectral changes. L-arginine L-glutamate salt caused a shift in position for many amide peaks (as shown in the boxes in Fig. 1A) and further reduced the signal intensity of amide peaks that were already weak. As a result, many peaks in the peripheral region of the spectrum diminished or disappeared (Fig. 1A), indicating that L-arginine L-glutamate salt increased the structural inhomogeneity of PB2 627. In contrast, glycine did not move or quench most peripheral peaks (Fig. 1B).

### 3.3 Quantitative analysis

**Fig. 2:**
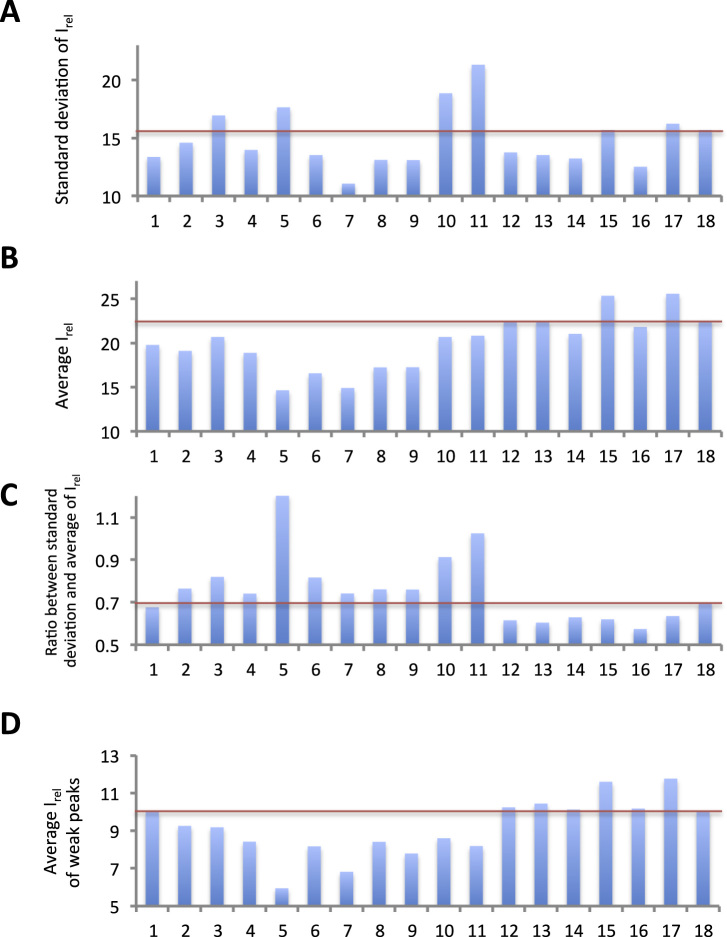
**Analysis of the homogeneity of I**_**rel**_**of backbone amide signals** The vertical axes indicate the standard deviation (A), average (B) and the ratio of the standard deviation to the average (C) of I_rel_, respectively. The vertical axis of (D) indicates the average I_rel_ of the weakest 50 peaks. Lane 1: 1,4-diaminobutane; lane 2: 1,5-diaminopentane; lane 3: CHAPS; lane 4: glycerol; lane 5: trehalose; lane 6: Na_2_SO_4_; lane 7: NDSB195; lane 8: NDSB201; lane 9: hydroxyectoine; lane 10: (L-arginine)_2_SO_4_; lane 11: L-arginine L-glutamate salt; lane 12: TMAO; lane 13: betaine; lane 14: L-proline; lane 15: L-α-alanine; lane 16: β-alanine; lane 17: glycine; lane 18: no additive. Red bars indicate the values obtained in the absence of additives.

**Fig. 3:**
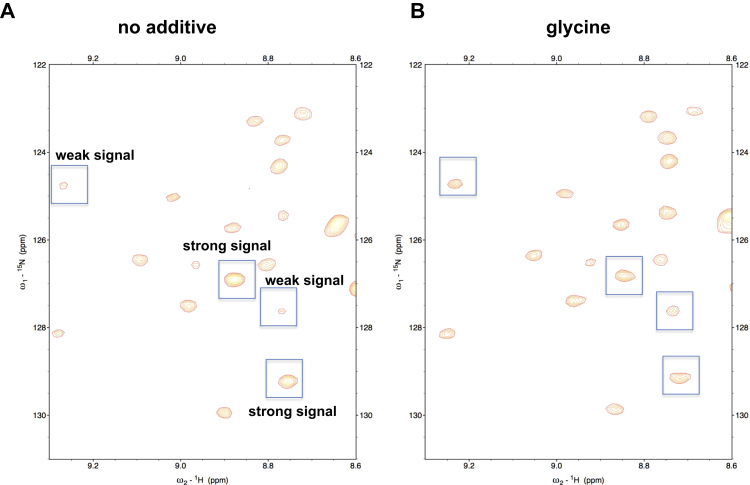
**Comparison of amide peaks, with or without glycine** A region in the 2D ^1^H–^15^N HSQC spectrum (A) without or (B) with glycine. Boxed peaks showed different intensities between (A) and (B).

To quantitatively analyze the inhomogeneity of peak intensities, we picked 210 backbone signals that were recognized as peaks by Sparky 3. While the expected number of backbone amide signals was 215, it is quite difficult to pick a complete set of backbone amide signals due to extensive signal overlaps and the existence of numerous weak peaks that could not be clearly distinguished from noise. Thus, we anticipated that PB2 627 contains structural polymorphisms. We calculated the ratio of the signal intensities of individual resonances to the average noise, I_rel_, in order to measure the S/N ratios of the individual resonances, as well as their standard deviations to measure inhomogeneities in the signal intensities. We found that 11 additives decreased the standard deviation of I_rel_ for the 210 peaks compared with the no-additive condition (Fig. 2A). Furthermore, we found that the average I_rel_ decreased in many cases (Fig. 2B), suggesting that the decrease in standard deviation may be due to a lower average I_rel_ value. We therefore calculated the ratio of the standard deviation to the average and found that the ratio increased in the presence of 10 additives, compared with the no-additive condition (Fig. 2C), suggesting that these compounds increased the inhomogeneity of I_rel_. The ratio increased particularly in the presence of CHAPS, trehalose, Na_2_SO_4_, (L-arginine)_2_SO_4_, and L-arginine L-glutamate salt. In contrast, neutral amino acids and derivatives such as TMAO, betaine, L-proline, L-α-alanine, β-alanine, and glycine reduced the ratio, suggesting that these compounds increased the homogeneity of the signal intensities. The average I_rel_ values of the 50 weakest peaks were greater under the neutral-amino acid and derivative conditions than under the no-additive condition (Fig. 2D). The values were markedly high under the L-α-alanine and glycine conditions. These results suggested that the increase in the I_rel_ of weak signals contributed to the decreased ratio of the standard deviation to the average. The improvement observed in the quantitative analysis was confirmed qualitatively in the spectra (Fig. 3).

Next, we asked whether increasing concentration of L-α-alanine and glycine further improved the quality of the spectra (Supplementary Table 2). Average I_rel_ for the 210 amide peaks was greater under the condition with 0.5 M L-α-alanine than under the conditions with 0 and 1.0 M L-α-alanine. Similarly, average I_rel_ for the 210 peaks was the greatest under the condition with 0.5 M glycine compared with the conditions with the other glycine concentrations. In contrast, the more the concentration of the additive amino acid was, the smaller the ratio between the standard deviation and average of I_rel_ of the amide peaks was.

### 3.4 NMR profiles

**Fig. 4:**
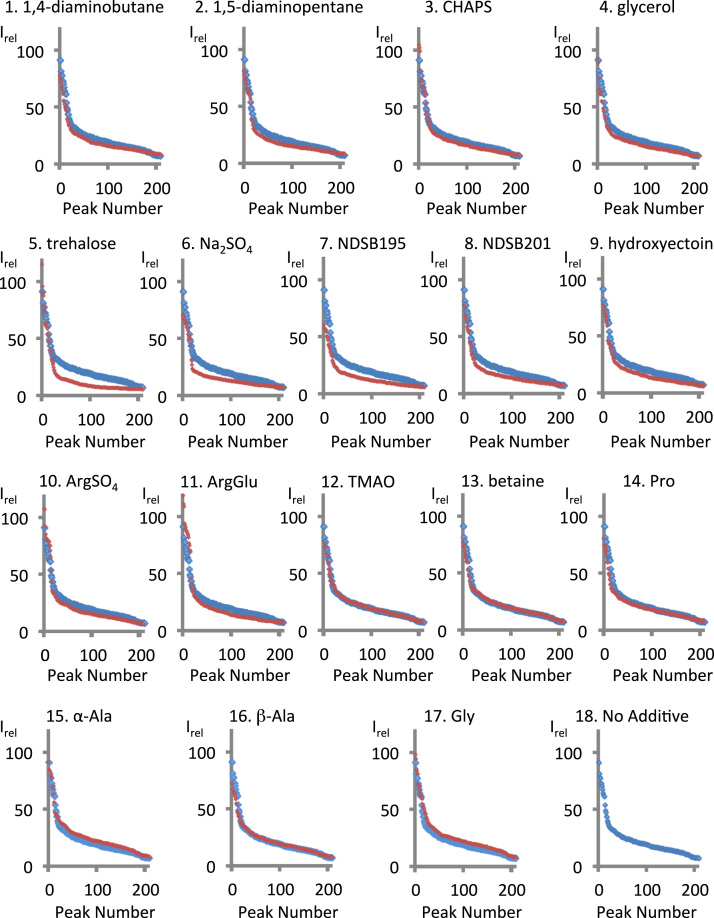
**NMR-profile of PB2 627, with or without additives** The NMR profiles of the 18 conditions tested in Fig. 2 are shown. The longitudinal and horizontal axes show the I_rel_ values and peak numbers, respectively. The conditions are numbered identically as done in Fig. 2. Plots for each individual additive conditions are shown with red dots, and data points obtained with the no-additive condition are shown with blue dots as a reference.

NMR profiling is useful for assessing the distribution of I_rel_ values of proteins [26]. We profiled the I_rel_ values of backbone amide protons from the ^1^H–^15^N HSQC spectra (Fig. 4). Most plots in the NMR-profiles of 1,4-diaminobutane, 1,5-diaminopentane, glycerol, Na_2_SO_4_, NDSB 195, NDSB 201, and hydroxyectoine (red data points) were lower than those in the NMR-profile under the no-additive condition (blue data points). Between peak numbers ~ 15 to 210, the plots for CHAPS, trehalose, (L-arginine)_2_SO_4_, and L-arginine L-glutamate salt showed lower I_rel_ values than observed with the no-additive condition, whereas the I_rel_ values tended to be higher than observed with the no-additive condition between peak numbers 1 to ~ 15. With L-α-alanine and glycine, most plots for peak numbers between ~ 15 and 210 showed higher I_rel_ values than observed with the no-additive condition, whereas the plots under these additive conditions and no-additive condition were superimposable for peak numbers 1 to ~ 15. The plots for β-alanine showed lower I_rel_ values compared to the no-additive control between peak numbers 1 to ~ 15 and were superimposable between peak numbers ~ 15 to 210. Similar tendencies are observed in the cases of TMAO, betaine, and L-proline.

## 4 Discussion

We expressed and purified ^15^N-labeled PB2 627, which has a nuclear localization signal and a C-terminal RNA-binding domain. The ^1^H–^15^N HSQC data indicated that the protein was folded, and was not aggregated in solution. This observation is consistent with the previous success in crystallizing PB2 627 [11], [12], [13], a process that would have required a stably folded protein. However, peaks due to backbone amides varied in intensity, suggesting that transient structural changes, structural polymorphisms, local denaturation, and/or transient, non-specific interactions occurred. Therefore, we collected spectral data in the presence of various additives that may change the structural properties of PB2 627 and improve the spectra. Consequently, we found that TMAO, betaine, L-proline, L-α-alanine, β-alanine, and glycine improved the spectral quality by increasing the homogeneity of signal intensities, whereas the other compounds did not. In addition, L-α-alanine and glycine markedly improved the average ratio of intensity of individual signals to noise levels (i.e., the I_rel_ values).

A natural question is why these compounds improved the quality of the spectral data. These compounds are osmolytes, which are naturally occurring chemical chaperones [15], [20], [22] that enhance hydration of partially denatured proteins to prevent complete denaturation and oligomerization/aggregation [27], [28]. Thus, the ability of these additives to homogenize the structural state of PB2 627 and improve the spectral quality was not surprising. The NMR profiles showed that L-α-alanine and glycine improved I_rel_ values, except for those with small peak numbers (i.e. those with high I_rel_ values), which suggests the occurrence of decreased structural polymorphisms and/or line broadening due to local motion over a time scale ranging from microseconds to milliseconds. In contrast, I_rel_ values with small peak numbers did not change compared with those observed under the no-additive condition, suggesting that the mobility of the unstructured regions of PB2 627 was unchanged. Although most amide peaks did not move upon the addition of glycine, a few peaks moved upfield, as shown in Fig. 1B, which may suggest that hydrogen bonds were weakened. This, in turn, may suggest that transient interactions between PB2 627 monomers or between PB2 627 and solutes decreased upon the addition of glycine, with increased hydration. Indeed, the quality of the spectrum of PB2 627 decreased when we increased the concentration of PB2 627, which suggested non-specific interaction between monomers of PB2 627 (Supplementary Table. 3). L-α-alanine and glycine have low carbon contents, each with no more than three carbon atoms, and have multiple hydrophilic groups. In the cases of TMAO, betaine, L-proline, and β-alanine, the I_rel_ values with small peak numbers decreased compared with those observed in the absence of additive, which suggests that these compounds decreased the mobility of the unstructured regions. Although these compounds did not improve the average I_rel_, it is intriguing that they improved the ratio between the standard deviation and the average of I_rel_ through an alternative mechanism to that of L-α-alanine and glycine. These compounds contain 3–5 carbon atoms with 1 or 2 hydrophilic group(s); thus, they are smaller than detergents and sugars. Remarkably, the effects of L-α-alanine and β-alanine were dissimilar, although these amino acids have identical chemical formulas. We note that these isomers differ in terms of water solubility and other characteristics, despite their high structural similarity.

No other additives tested improved the quality of the spectral data. In particular, polyols and sugars such as trehalose and sucrose resulted in extensive degradation of the spectra. This result was perhaps due to their higher molecular weight or the presence of aldehyde groups in sugars. In addition, the bulky amino acid hydroxyectoine, as well as compounds with relatively large hydrophobic groups, including 1,5-diaminopentane, CHAPS, β-octylglucoside, and NDSBs, did not improve the quality of the spectra. Notably, hydrophobic molecules may either stabilize or destabilize proteins by interacting with hydrophobic patches on the surface, or with the hydrophobic core, respectively [27], [28]. Thus, the data may suggest that these hydrophobic additives mainly interacted with the hydrophobic core of PB2 627, presumably because hydrophobic patches on the PB2 627 surface are relatively small. In fact, there are only 2 aromatic amino acids on the surface: F76 and Y170 (Supplementary Fig. 4). Accordingly, L-arginine, which is frequently used to stabilize hydrophobic groups on protein surfaces [29], also degraded the spectra. In the presence of L-arginine, the I_rel_ of peak numbers ranging from 1 to ~ 15 increased, suggesting that the extent of mobility of the unstructured regions increased. It has been reported that L-arginine L-glutamate salt is effective in improving NMR spectra over a concentration range of 0.05–0.77 M [15], [16]. High concentration of salts causes decreased S/N ratios with modern cryoprobes, so the possibility remains that lower concentrations of L-arginine L-glutamate and other salts provide a different result. The inorganic salt Na_2_SO_4_ also degraded the quality of spectral data, although SO_4_^2-^ appears early in the Hofmeister series. This may be because SO_4_^2-^ is not electrically neutral, as is L-glutamate, which also did not improve the quality of the NMR data.

Other biophysical screening methods used to optimize solution conditions, such as dynamic light scattering and the Thermofluor assay, may be easier than the present NMR-based method to check the effects of additive compounds on propensities for aggregation and thermostability, respectively. It is, however, quite difficult to study the properties of local structural dynamics and polymorphisms by those biophysical methods, which NMR sensitively detects.

The HSQC spectrum of PB2 627 showed well-dispersed amide signals, suggesting that global folding is stable in aqueous solution, in line with its apparent crystallizability [11], [12], [13]. However, the intensities of backbone amide peaks were quite inhomogeneous, indicating that the protein structure and dynamic properties of PB2 627 are inhomogeneous. Therefore, we collected NMR data in the presence of various additives to examine whether those compounds homogenize the structural state of PB2 627. We compared the resulting spectra using the ratio between the standard deviation and average of I_rel_ of the amide peaks, which represents a novel approach for assessing NMR data quality. Based on this analysis, we found that small osmolytes could improve the quality of NMR spectra by increasing homogeneity of the structural states of PB2 627. Among these compounds, L-α-alanine and glycine remarkably improved the I_rel_ of weak signals, which is advantageous for 3-dimensional measurements. The NMR profiles indicated that these additives influenced the dynamic properties of PB2 627. In contrast, hydrophobic additives did not improve the quality of the spectra, presumably because such compounds perturbed the folding of PB2 627 by interacting with its hydrophobic core. These results will facilitate future structural and dynamics studies of PB2 627 in solution, as well as drug design targeting PB2 627.

It has been reported that L-α-alanine and glycine stabilize proteins. Matthews and Leatherbarrow reported that glycine up to 2 M concentration increased the thermal stability of the folded structure of lysozyme [30]. The mechanism by which L-α-alanine and glycine stabilize protein structures is through hydration of protein molecules [20]. The hydration properties of these amino acids are common with other stabilizing chemicals such as glycerol and L-arginine L-glutamate salt. Thus, we anticipate that these small amino acids could be generally applied to stabilize other proteins.

Our results and methodologies will also help develop NMR, chemical, pharmacological, and quality-control techniques for analyzing protein drugs, which are now increasingly used as therapeutics. In addition, our methods will also help identify, evaluate, or develop stabilizing agents for such protein drugs, which are generally much less stable than small molecules.
