# Supplementary material for: NMR spectra of PB2 627, the RNA-binding domain in influenza A virus RNA polymerase that contains the pathogenicity factor lysine 627, and improvement of the spectra by small osmolytes
Source: Biochem Biophys Rep. 2017 Sep 20;12:129–34. doi: 10.1016/j.bbrep.2017.09.003 (PMC5645118; doi:10.1016/j.bbrep.2017.09.003)
Supplement: S — upplementary material [file mmc1.docx]

We declare no conflict of interest.
