# Supplementary material for: NMR spectra of PB2 627, the RNA-binding domain in influenza A virus RNA polymerase that contains the pathogenicity factor lysine 627, and improvement of the spectra by small osmolytes
Source: Biochem Biophys Rep. 2017 Sep 20;12:129–34. doi: 10.1016/j.bbrep.2017.09.003 (PMC5645118; doi:10.1016/j.bbrep.2017.09.003)
Supplement: Supplementary file 2 — Supplementary material [file mmc2.docx]

**Supplementary Figure legends**

**Supplementary Figure 1: Purification of PB2 627**

SDS-PAGE analysis of PB2 627 at each purification step

**Supplementary Figure 2: ^1^H-^15^N HSQC spectra of PB2 627**

Spectra are contoured at (A) 1.7 e+04 and (B) 2.9 e+04.

**Supplementary Figure 3: ^1^H-^15^N HSQC spectra of PB2 627 in the presence of additives**

Spectra were measured in the presence of (A) no additive, (B) 0.5 M glycine, (C) 4 % glycerol, (D) 0.5 M β-alanine, and (E) 0.2 M L-arginine L-glutamate salt.

**Supplementary Figure 4: Aromatic amino acids in PB2 627**

Space-filling model of PB2 627. Tyrosine, phenylalanine, and tryptophan residues are highlighted in cyan, green, and magenta, respectively. Tryptophan residues are not observed in this view.
