# Supplementary material for: NMR spectra of PB2 627, the RNA-binding domain in influenza A virus RNA polymerase that contains the pathogenicity factor lysine 627, and improvement of the spectra by small osmolytes
Source: Biochem Biophys Rep. 2017 Sep 20;12:129–34. doi: 10.1016/j.bbrep.2017.09.003 (PMC5645118; doi:10.1016/j.bbrep.2017.09.003)
Supplement: Supplementary file 3 — Supplementary material [file mmc3.pdf]

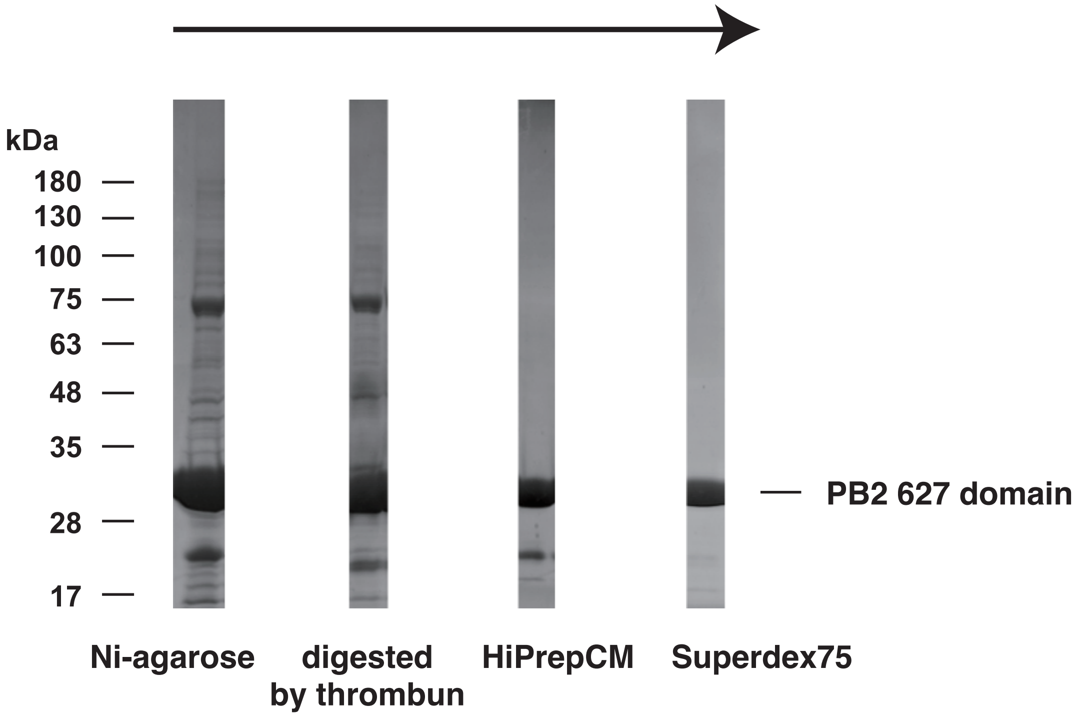

Supplementary Figure 1

A

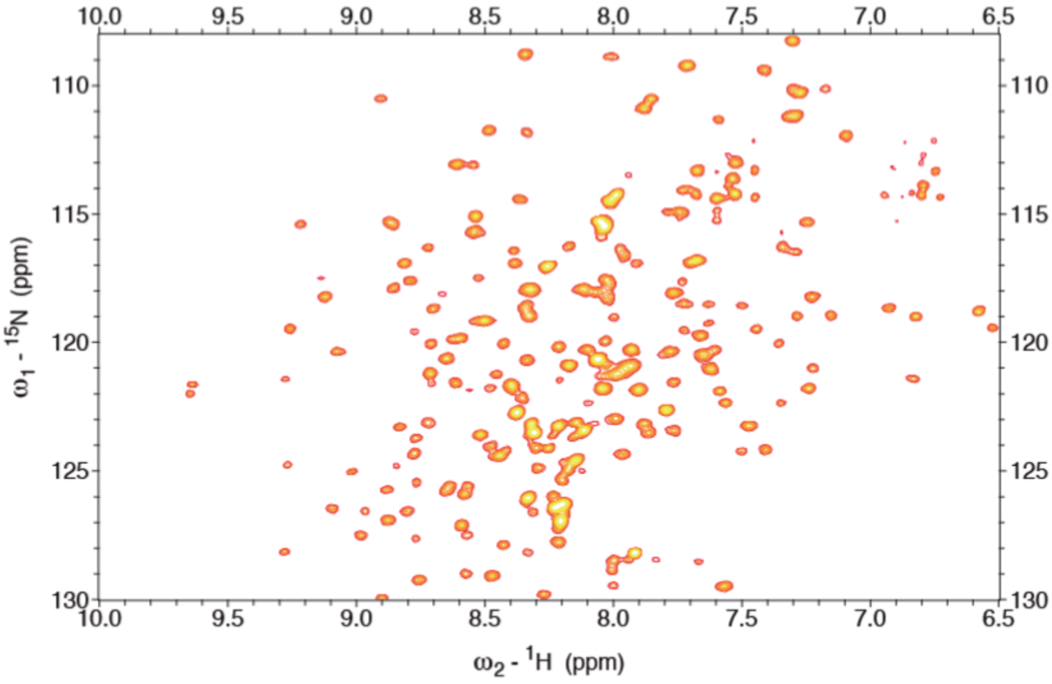

B

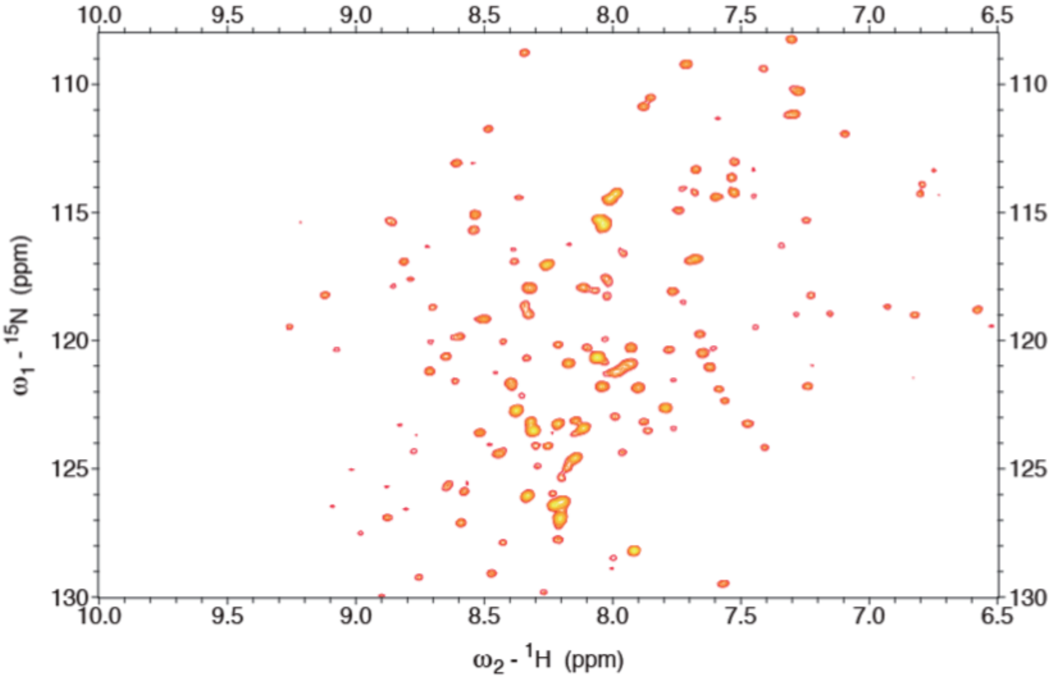

Supplementary Figure 2

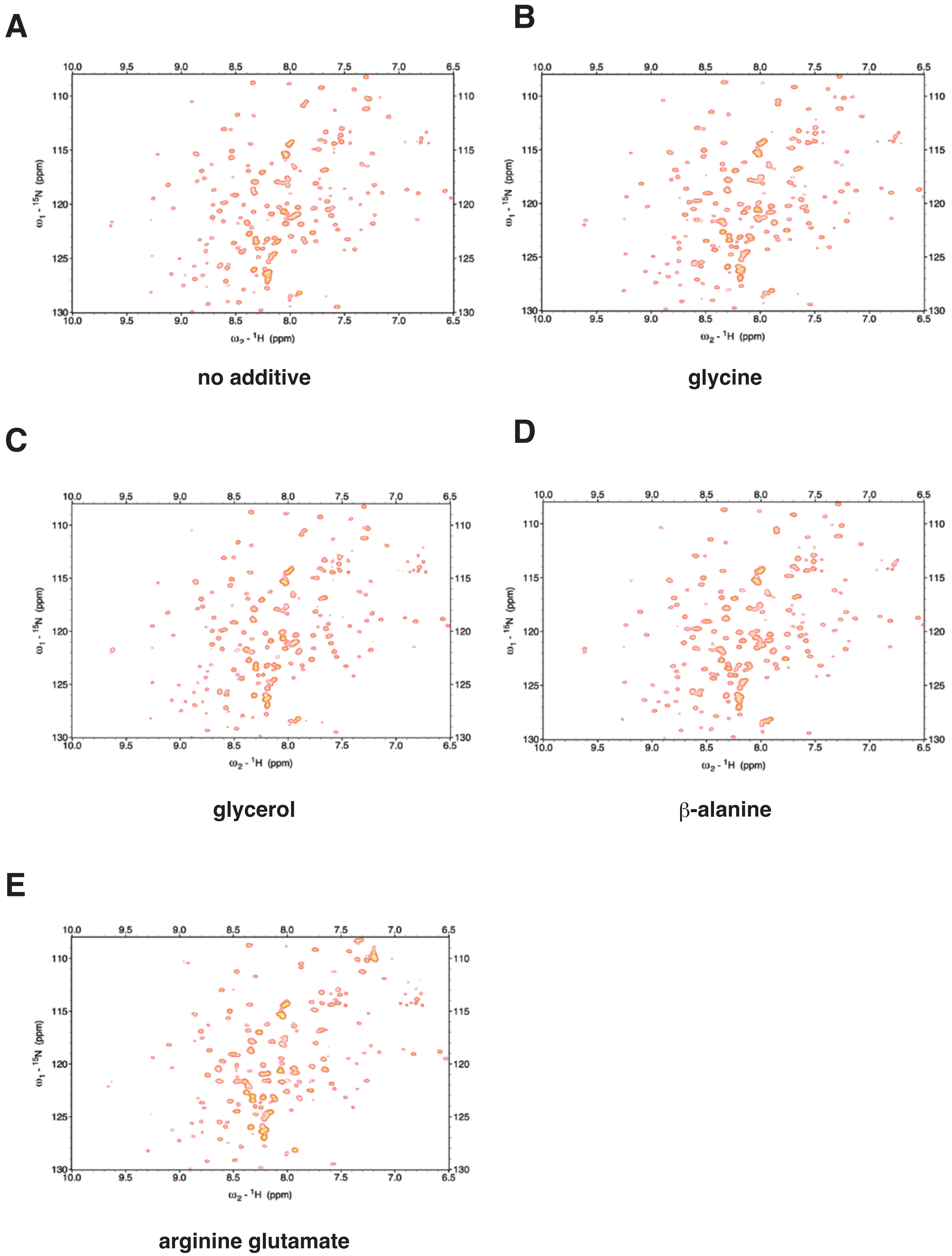

Supplementary Figure 3

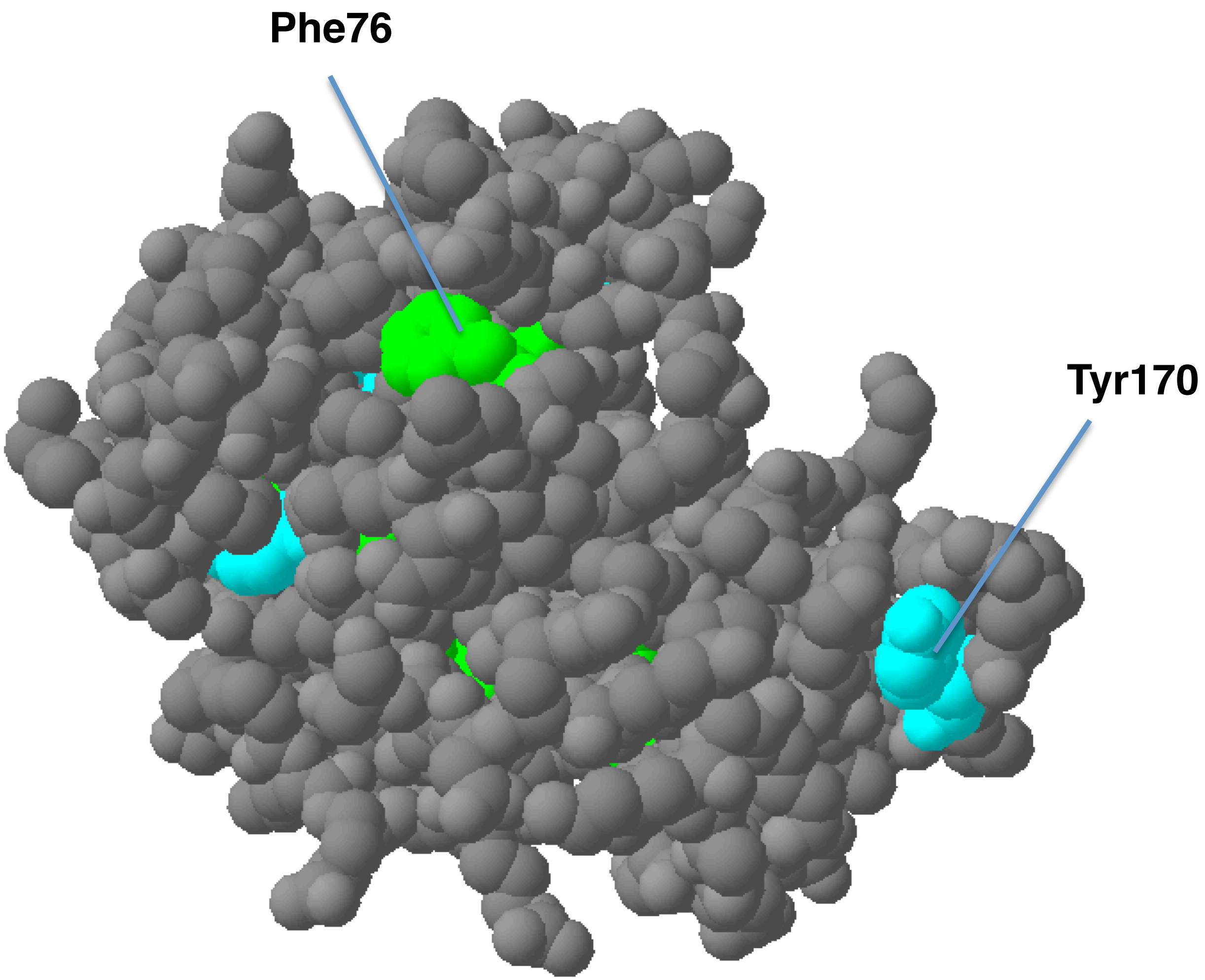

Supplementary Figure 4
