# Supplementary material for: NMR spectra of PB2 627, the RNA-binding domain in influenza A virus RNA polymerase that contains the pathogenicity factor lysine 627, and improvement of the spectra by small osmolytes
Source: Biochem Biophys Rep. 2017 Sep 20;12:129–34. doi: 10.1016/j.bbrep.2017.09.003 (PMC5645118; doi:10.1016/j.bbrep.2017.09.003)
Supplement: Supplementary file 4 — Supplementary material [file mmc4.docx]

**Supplementary Table 1. Additives**

***Additive*** ***Reference***

1. 1,4-diaminobutane [21]

2. 1,5-diaminopentane [21]

3. CHAPS [15]

4. glycerol [15, 28]

5. trehalose [15, 28]

6. Na_2_SO_4_ [15]

7. NDSB 195 [17, 31]

8. NDSB 201 [17, 31]

9. hydroxyectoine [32]

10. (L-arginine)_2_SO_4_ [19]

11. L-arginine L-glutamate salt [16]

12. TMAO [15, 28]

13. betaine [15, 28]

14. L-proline [15, 28]

15. L-α-alanine [15, 28]

16. β-alanine [15, 28]

17. glycine [15, 28]

***Additives that denatured PB2 627***

1. sucrose [15, 28]

2. β-octylglucoside [15]

3. NDSB 256 [33]

**Supplementary Table 2. Effects of higher concentration of additives**

|  | No additive | 0.5 M Ala | 1.0 M Ala |
| --- | --- | --- | --- |
| Average I_rel_ | 17.0 | 22.3 | 20.7 |
| Ratio between standard deviation and average I_rel_ | 0.681 | 0.645 | 0.617 |
| Average I_rel_ of weak peaks | 5.47 | 7.65 | 7.60 |

|  | No additive | 0.5 M Gly | 1.0 M Gly | 1.5 M Gly |
| --- | --- | --- | --- | --- |
| Average I_rel_ | 17.0 | 25.5 | 23.8 | 20.0 |
| Ratio between standard deviation and average I_rel_ | 0.681 | 0.643 | 0.629 | 0.589 |
| Average I_rel_ of weak peaks | 5.47 | 8.02 | 7.86 | 7.87 |

**Supplementary Table 3. Effect of concentration of PB2 627**

| Concentration | 25 μM | 500 μM |
| --- | --- | --- |
| Ratio between standard deviation and average I_rel_ | 0.695 | 0.804 |

**Supplementary References**

[31] L. Vuillard, C. Braun-Breton, T. Rabilloud. Non-detergent sulphobetaines: a new class of mild solubilization agents for protein purification. Biochem. J. 305 (1995) 337-343.

[32] M. Kanapathipillai, G. Lentzen, M. Sierks, C.B. Park. Ectoine and hydroxyectoine inhibit aggregation and neurotoxicity of Alzheimer's beta-amyloid. FEBS Lett. 579 (2005) 4775-4780.

[33] M.E. Goldberg, N. Expert-Bezancon, L. Vuillard, T. Rabilloud. Non-detergent sulphobetaines: a new class of molecules that facilitate *in vitro* protein renaturation. Fold Des. 1 (1996) 21-27.
